# The Essential Genome of *Escherichia coli* K-12

**DOI:** 10.1128/mBio.02096-17

**Published:** 2018-02-20

**Authors:** Emily C. A. Goodall, Ashley Robinson, Iain G. Johnston, Sara Jabbari, Keith A. Turner, Adam F. Cunningham, Peter A. Lund, Jeffrey A. Cole, Ian R. Henderson

**Affiliations:** a Institute of Microbiology and Infection, University of Birmingham, Birmingham, United Kingdom; b Discuva Ltd., Cambridge, United Kingdom; National University of Singapore and Genome Institute of Singapore; Nanyang Technological University

**Keywords:** *Escherichia coli*, TraDIS, genomics, tn-seq

## Abstract

Transposon-directed insertion site sequencing (TraDIS) is a high-throughput method coupling transposon mutagenesis with short-fragment DNA sequencing. It is commonly used to identify essential genes. Single gene deletion libraries are considered the gold standard for identifying essential genes. Currently, the TraDIS method has not been benchmarked against such libraries, and therefore, it remains unclear whether the two methodologies are comparable. To address this, a high-density transposon library was constructed in *Escherichia coli* K-12. Essential genes predicted from sequencing of this library were compared to existing essential gene databases. To decrease false-positive identification of essential genes, statistical data analysis included corrections for both gene length and genome length. Through this analysis, new essential genes and genes previously incorrectly designated essential were identified. We show that manual analysis of TraDIS data reveals novel features that would not have been detected by statistical analysis alone. Examples include short essential regions within genes, orientation-dependent effects, and fine-resolution identification of genome and protein features. Recognition of these insertion profiles in transposon mutagenesis data sets will assist genome annotation of less well characterized genomes and provides new insights into bacterial physiology and biochemistry.

## IMPORTANCE

Incentives to define lists of genes that are essential for bacterial survival include the identification of potential targets for antibacterial drug development, genes required for rapid growth for exploitation in biotechnology, and discovery of new biochemical pathways. To identify essential genes in *Escherichia coli*, we constructed a transposon mutant library of unprecedented density. Initial automated analysis of the resulting data revealed many discrepancies compared to the literature. We now report more extensive statistical analysis supported by both literature searches and detailed inspection of high-density TraDIS sequencing data for each putative essential gene for the *E. coli* model laboratory organism. This paper is important because it provides a better understanding of the essential genes of *E. coli*, reveals the limitations of relying on automated analysis alone, and provides a new standard for the analysis of TraDIS data.

## INTRODUCTION

There are many incentives to define lists of genes that are either essential for bacterial survival or important for normal rates of growth. Essential genes of bacterial pathogens may encode components of novel biochemical pathways or potential targets for antibacterial drug development. Disruption of genes required for rapid growth results in strains handicapped for exploitation in biotechnology. Conversely, normal growth of mutants defective in genes previously expected to be essential could reveal unexpected parallel biochemical pathways for fulfilling the essential function.

Multiple attempts have been made to generate definitive lists of essential genes, but there are still many discrepancies between studies even for the model bacterium *Escherichia coli* strain K-12. Two general approaches have been used: targeted deletion of individual genes, as in the Keio collection of mutants (1), and random mutagenesis (2, 3). Data from several studies using different mutagenesis strategies have yielded inconsistent data and hence conflicting conclusions. Transposon-directed insertion site sequencing (TraDIS) is one of several high-throughput techniques that combine random transposon mutagenesis with sequencing of the transposon junctions in high-density mutant libraries (4–7). Since its inception in 2009, this high-throughput method has been applied to a range of biological questions (4, 8–15). Here, in order to resolve outstanding conflicts, we report the use of this approach to identify the essential genes of *E. coli* K-12 strain BW25113, a well-studied model organism for which a complete gene deletion library is available (1).

A confounding factor in determining the “essentiality” of a gene is the definition of an essential gene. Complete deletion of an essential gene results, by definition, in a strain that cannot be isolated following growth. However, it is well-known that certain genes are required for growth under specific environmental and nutritional conditions. Such genes can be considered conditionally essential. For the purposes of this study, we define a gene as essential if the transposon insertion data reveal that the protein coding sequence (CDS), or a portion of the CDS, is required for growth under the conditions tested here. To aid our analysis, we developed a statistical model that included corrections for both gene length and genome length in order to decrease false-positive identification of essential genes.

An additional challenge with defining essentiality in high-throughput studies is an overreliance on automated analysis of the data. For example, a consequence of relying only on quantification of the number of unique insertions within a gene is that genes with essential regions will be missed. If only part of a gene encodes the essential function, it should be possible to isolate viable mutants with transposon insertions in nonessential regions of the coding sequence (2). Conversely, reliance on statistical analysis alone can also lead to overestimation of the number of essential genes. This is a common result from insertion sequencing analysis (16). A low number of transposon insertion events within a gene, which fall below the statistical cutoff threshold, can be due to inaccessibility of the gene to transposition because of extreme DNA structure, exclusion by DNA-binding proteins, polarity effects due to insertion in a gene upstream of a cotranscribed essential gene, and location of the gene close to the replication terminus (17). The most frequent reason for a low number of insertions is that the product of the disrupted gene is required for normal rates of growth under the conditions tested. In the current study, to minimize the possibility of incorrectly designating genes as essential or contributing to fitness, we have supported our statistical analysis with a gene-by-gene inspection of the insertion distribution within each individual gene.

## RESULTS AND DISCUSSION

### Sequencing of a mini-Tn*5* transposon insertion library in *E. coli* strain BW25113.

**FIG 1:**
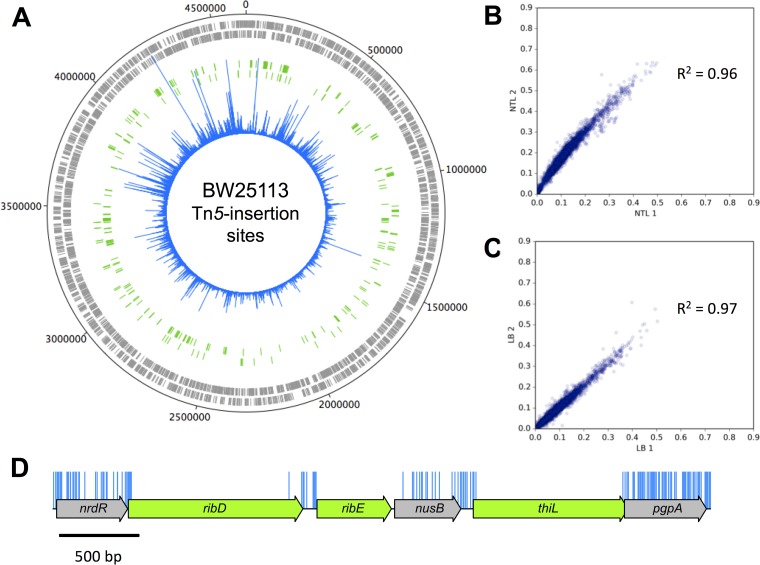
Genome-wide transposon insertion sites mapped to *E. coli* strain BW25113**.** (A) Frequency and location of transposon junction sequences from a mini-Tn*5* transposon library in strain BW25113, mapped to the BW25113 genome (CP009273.1). The outermost track marks the BW25113 genome in base pairs starting at the annotation origin. The next two inner tracks correspond to sense and antisense CDS, respectively (gray), followed by two inner tracks depicting the essential genes identified by TraDIS on the sense and anti-sense strands, respectively (green). The innermost circle (blue) corresponds to the frequency and location of transposon insertion sequences mapped successfully to the BW25113 genome after identification of a transposon sequence. This figure was created using DNAPlotter. (B and C) Correlation coefficients of gene insertion index scores for two sequenced technical replicates of the input transposon library (TL1 and TL2) (B) and following growth in LB (LB1 and LB2) (C). (D) Representation of transposon insertion points across a portion of the *E*. *coli* K-12 BW25113 genome (blue), showing essential genes (green) and nonessential genes (gray). Blue bars correspond with transposon insertion sites along the genome and have been capped at a frequency of 1.

We have used a modified method to obtain TraDIS data for a transposon mutant library of *E. coli* K-12 strain BW25113 (4, 9). The BW25113 strain was chosen because it is the parent strain for the Keio collection of deletion mutants and ideal for a direct comparison between data sets. A mini-Tn*5* transposon with a chloramphenicol resistance cassette was transformed into competent cells and grown overnight on selective medium. Individual colonies were pooled to construct the initial library, estimated to consist of approximately 3.7 million mutants. An Illumina MiSeq system was used to obtain TraDIS data from two independent DNA extracts of the transposon library (TL), designated TL1 and TL2 (Table 1). Raw data were checked for the presence of an inline index barcode to identify independently processed samples (Table 1). This resulted in 4,818,864 sequence reads from TL1 and 6,189,409 from TL2. After verification of the presence of a transposon sequence and removal of poor-quality data or short sequence reads, 3,891,339 (80.75%) and 4,387,970 (70.89%) sequence reads, respectively, were mapped successfully to the *E. coli* K-12 BW25113 genome (accession no. CP009273.1) (Table 1). The distribution of insertion sites covers the full length of the genome (Fig. 1A). There was a high correlation coefficient of 0.96 between the samples (Fig. 1B). The data were therefore combined to give a total of 8,279,309 sequences that were mapped to 901,383 unique insertion sites throughout the genome. Of the 8,279,309 mapped sequences, 199,557 were represented by a single read. Similar numbers of insertions, 481,360 and 480,072, were found for both orientations of the transposon. The high density of unique insertion sites resulted in an average of one insertion every 5.14 bp and a median distance between insertions of 3 bp. An example is shown in Fig. 1D.

**TABLE 1:** Parameters for TraDIS data set derived from the *E. coli* K-12 strain BW25113 transposon library

Condition	No. of sequence reads with matching inline barcode	No. of mapped sequence reads (% of the raw data)	No. of genome-wide insertion sites
TL1	4,818,864	3,891,339 (80.75)	500,476
TL2	6,189,409	4,387,970 (70.89)	817,011
TL combined	11,008,273	8,279,309	901,383
LB1	5,908,163	5,201,711 (88.04)	400,009
LB2	6,403,324	5,382,477 (84.06)	419,660
LB combined	12,311,487	10,584,188	595,233

### Identification of putative essential genes by TraDIS.

To determine whether a gene was essential or nonessential, the numbers of insertions per CDS were quantified. CDS is defined as the protein coding sequence of a gene, inclusive of the start and stop codons. To normalize for gene length, the number of unique insertion points within the CDS was divided by the CDS length in bases. This value was termed the insertion index score and has been used previously as a measure of essentiality (4, 8, 9, 18), given a sufficiently dense library (19).

The frequency distribution of the insertion index scores was bimodal (see Fig. S1 in the supplemental material), as previously shown by others (2). We assume that genes associated with the left mode (any data to the left of the trough in Fig. S1), which have a low number of transposon insertions, are either essential for survival or genes that, when disrupted, confer a very severe fitness cost (Fig. 1D). The second mode is associated with genes with considerably more insertions; these genes are deemed nonessential (Fig. 1D). Based on inspection of the distributions, an exponential distribution model was fitted to the mode that includes essential genes, and a gamma distribution model was fitted to the nonessential mode. For a given insertion index score, the probability of belonging to each mode was calculated, and the ratio of these values was termed the log likelihood score. A gene was classified as essential if its log likelihood score was less than log_2_(12) and was therefore 12 times more likely (see Materials and Methods) to belong to the essential mode than to the nonessential mode. Using this approach, sufficient insertions were found in 3,793 genes for them to be classed as nonessential, 162 genes were situated between the two modes and classed as unclear, and 358 genes in the mutant library were identified as essential (Table S1).

10.1128/mBio.02096-17.2 FIG S1  Frequency distribution of insertion index scores. The insertion index score for each coding sequence was calculated as the number of insertions per CDS divided by the CDS length in base pairs to normalize for gene length. The frequency of insertion index scores was plotted for both TL data (A) and LB data (B), and both followed a bimodal distribution. An exponential distribution model was fitted to the left mode that includes essential genes (red), and a gamma distribution model was fitted to the right, nonessential mode (blue). For a given insertion index score, the probability of belonging to each mode was calculated, and the ratio of these values was the log likelihood score. A gene was classified as essential if its log likelihood score was less than log_2 _12 and was therefore 12 times more likely to belong to the red mode than the blue mode. Download FIG S1, PDF file, 0.02 MB. Copyright © 2018 Goodall et al. 2018 Goodall et al. This content is distributed under the terms of the Creative Commons Attribution 4.0 International license.

10.1128/mBio.02096-17.4 TABLE S1  Essential genes identified by TraDIS as essential. Download TABLE S1, XLSX file, 0.2 MB. Copyright © 2018 Goodall et al. 2018 Goodall et al. This content is distributed under the terms of the Creative Commons Attribution 4.0 International license.

**FIG 2:**
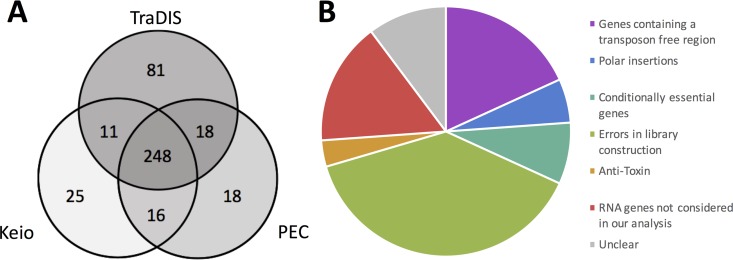
Comparison of essential gene data from various sources and examples of insertion profiles overlooked by automated statistical analysis of insertion index scores. (A) Putative essential genes identified using TraDIS were compared to existing essential gene data. A three-way comparison between the Keio collection of single gene knockouts, the online Profiling of the *E. coli* Chromosome (PEC) database, and our transposon insertion sequencing data identified 248 essential genes that were common to all three data sets. (B) The outlying genes of the Venn diagram, excluding those unique to our TraDIS data set, were inspected to understand the source of discrepancy between data sets. Genes were grouped into the overarching categories of “genes containing a transposon-free region,” “antitoxin,” “polar insertions,” “conditionally essential genes,” and “errors in library construction.” Genes not included in our analysis or that remain unclear are shown in red or gray, respectively.

The 358 putative essential genes identified in the TL data were compared to the essential genes as defined by the Keio collection and the Profiling of the *E. coli* Chromosome (PEC) database (1, 2). This comparison revealed 248 genes (59.5%) that were common to all three data sets (Fig. 2A and Table S2). This agreement between all three data sets strongly supports the hypothesis that these genes are essential so they were not investigated further. An additional 169 genes were identified as potentially essential in only one or two of the data sets. These genes comprise 16 genes in the Keio and PEC lists that were not identified by our analysis, 25 exclusive to Keio, 18 exclusive to PEC, and 11 and 18 that overlapped between our method and Keio or PEC, respectively (Fig. 2A). However, the largest subcategory of 81 genes is unique to our data set.

10.1128/mBio.02096-17.5 TABLE S2  Comparison of essential genes between data sets. Download TABLE S2, PDF file, 0.04 MB. Copyright © 2018 Goodall et al. 2018 Goodall et al. This content is distributed under the terms of the Creative Commons Attribution 4.0 International license.

### Statistical analysis of the transposon insertion density data.

Overestimation of the number of genes that are essential has been noted in studies using transposon insertion sequencing (16). In previous attempts to use statistical analysis to define an essential gene, a Poissonian model was used to derive a *P* value for an insertion-free region (IFR) of a given length against the null hypothesis that, by chance, no insertions occurred in that region. We refined this approach for two reasons. First, genomes are sequences of discrete sites: although a continuous Poisson model can provide an approximation to this structure, a naturally discrete picture is more representative of true genome structure. Second, unless corrections are applied for gene length or for the genome length, this method risks overestimating the total number of essential genes. This problem arises because the method implicitly considers only a single, small genomic region, giving the probability that no insertions will be found in a single region of a given base pair length. However, genes and genomes have many such regions that are effectively independent, so the genome-wide probability of observing a “false-positive” insertion-free region across the genome will be much higher.

To avoid this risk of overinterpretation of TraDIS data, we propose a new statistical approach, summarized in Text S1 and Fig. S2. First, we replaced the commonly used Poissonian model exp(−*x*/*f*) (for *x* consecutive bases without an insert, given inverse insertion density *f*; see reference 27 for further discussion of this) with a geometric model. This model gives the probability of seeing *k* “failures” (insertion-free sites) then a “success” (insertion event) in a string of independent trials as *P*(*k*) = ρ (1 − ρ)^*k*^, where ρ is the probability of a success (here, an insertion). The *P* value associated with a string of *L* sites being insertion-free is then $P=\overset{\infty }{\underset{k=L}{\sum }}P(k)$, an easily computable quantity. Next, to guard against false-positive results, we need to precisely state the statistic of interest and the corresponding null model. Under a null model of random, independent insertions, the three probabilities most pertinent here are those with which (i) a single length *L* region has no insertions; (ii) a gene of length *g* contains one or more insertion-free regions of length *L*; (iii) a genome of length *G* contains one or more insertion-free regions of length *L*. We used stochastic simulations of random insertions with given densities and genome lengths (Text S1) to compute these probabilities. These values then give *P* values for insertion-free region observations, correcting for gene and genome length. Specifically, *p*_gene_(*L*) is the probability of observing one or more insertion-free regions of at least length *L* in a model gene (of length *g* = 1,000 bp) by chance (ii), and *p*_genome_(*L*) is the probability of observing one or more insertion-free regions of at least length *L* in a full genome (of length *G* = 4.6 Mb) by chance (iii). The uncorrected *P* value (i) is that typically reported in other studies. Statistical analysis of our current data (901,383 inserts in a 4,631,469-bp genome) gives a corrected *p*_genome_ of 0.05 for *L* ≅ 75 bp and *p*_gene_ of 0.05 for *L* ≅ 36 bp (*p*_gene_ of 0.005 for *L* ≅ 47 bp). In other words, there is a probability of 0.05 that any insertion-free region of length 75 bp could appear anywhere in the genome by chance, and there is a probability of 0.005 that any insertion-free region of length 47 bp will occur anywhere in a gene of length 1,000 bp by chance. To our knowledge, this represents the first study with a confident and genome-wide corrected detection resolution (Fig. S2), and the closest yet to approaching the length of the smallest annotated gene in our reference genome (accession no. CP009273.1), which is 45 bp.

10.1128/mBio.02096-17.1 TEXT S1  Supplemental methods. Download TEXT S1, DOCX file, 0.1 MB. Copyright © 2018 Goodall et al. 2018 Goodall et al. This content is distributed under the terms of the Creative Commons Attribution 4.0 International license.

10.1128/mBio.02096-17.3 FIG S2  Simulation of random insertion events for rigorous statistics under the null model of random insertion. (A) The expected number of insertion-free regions (IFRs) of length *L* or above found in a genome of length *G* under the null model of *N* random noncoincident insertions. *G* and *N* are taken to correspond to the statistics in the studies plotted. This expectation is taken over 100 instances of the null model simulation. (B) The (related) probability of at least one IFR of length *L* or above occurring anywhere in the simulated genome. (C and D) The same calculation over a simulated gene of length *g* = 1,000, using 10^5^ simulation instances. Download FIG S2, TIF file, 0.1 MB. Copyright © 2018 Goodall et al. 2018 Goodall et al. This content is distributed under the terms of the Creative Commons Attribution 4.0 International license.

In checking for uniformity of insertion density across genomic regions, we found that the density of insertions around the terminus (taken as a region centered around *terABCD*) was slightly lower than the genomic average (a density of 0.142 in the surrounding 500-kb region, or 0.145 in the surrounding 1-Mb region, compared to a 0.195 average; Fig. 1A). This density change marginally increases the detection of false-positive essential genes in the vicinity of the terminus but still represents an unprecedented level of coverage.

### Resolution of conflicts between data sets.

A critical requirement for the validation of a list of essential genes is to explain why the statistical analysis of transposon insertion data failed to identify genes that the Keio library of deletion mutants and the PEC database identified as essential. We coupled statistical analysis and manual inspection of the data with literature searches to rationalize conflicting results. We find that many of the inconsistencies between data sets can be explained by different methodologies used, definitions of the term “essential,” and statistical approaches (Fig. 2B).

### Genes containing transposon-free regions.

**FIG 3:**
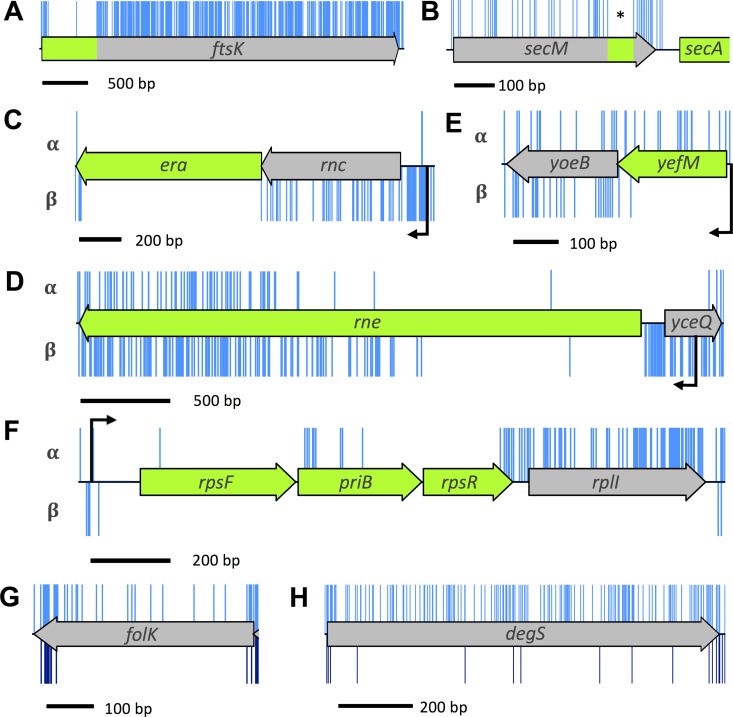
Insertion profiles of discrepant genes between data sets. (A) The *ftsK* gene codes for an essential protein in which only part of the protein is required for its essential function. Such genes have a high insertion index score and consequently would not have been identified by automated statistical analysis. (B) *secM* contains a window (indicated by an asterisk) of 66 bp in which there were no transposon insertions. This feature is discussed in the text. (C to F) Genes with transposon insertions in only one orientation. The α- and β-orientation of the transposon is depicted above and below the schematic representation of the CDS, respectively, and native promoters are shown in black. (G and H) Many transposon insertions were found along the full length of *folK* and *degS* (shown in blue above the schematic map of the figure). However, most of these insertion mutants were lost during outgrowth (below the schematic representation and shown in dark blue).

Manual inspection of the data revealed genes with transposon-free regions that were large enough to be identified as significant using the algorithm defined in the previous section. These IFRs do not necessarily report that a gene is essential; rather, they show that the insertions within these genes are sufficiently sparse that the IFR is unlikely to have occurred by chance. These genes fall loosely into two groups. The first group contains genes for which the 5′ regions are essential and contain no insertions. However, there are transposon insertions in the nonessential regions of these genes, such as *ftsK* (Fig. 3A and Table S3). FtsK is involved in correct segregation of the chromosome during division (20, 21); the N-terminal domain of FtsK contains four transmembrane passes and is required for localization of FtsK to the septum (22–24). There is substantial literature reporting the essential function of the N-terminal domain, consistent with our data (21, 22, 25). This is a common observation for insertion data and arises when only the function of the N terminus of the protein is required for viability (8). Initial analysis of transposon insertion data would lead to these genes being incorrectly classified as nonessential, but attempts to construct a deletion mutant would fail. Indeed, previous transposon sequencing experiments failed to identify the essential nature of some of these genes when relying on statistical analysis alone (9).

10.1128/mBio.02096-17.6 TABLE S3  Source of discrepancy between data sets. The outlying genes of the Venn diagram excluding those unique to our TraDIS data. Gene names used by the Keio collection are shown in parentheses. The Venn subcategory is indicated in the Venn group column as follows: K for Keio only, KP for Keio and PEC, KT for Keio and TraDIS (this study), P for PEC only, and PT for PEC and TraDIS. Download TABLE S3, PDF file, 0.03 MB. Copyright © 2018 Goodall et al. 2018 Goodall et al. This content is distributed under the terms of the Creative Commons Attribution 4.0 International license.

The second group contains genes with transposon insertion sites throughout the CDS but which have an IFR that passes the significance threshold for essentiality. For example, there is a small IFR within the coding sequence of *secM* of 66 bp (Fig. 3B and Table S3). The *secM* gene is located upstream of the essential gene *secA*. These genes are cotranscribed and also cotranslated, and *secM* is known to contain a translational stop sequence that interacts with the ribosomal exit tunnel to halt translation, acting as a translational regulator for *secA*. Specific mutations within the translational stop sequence are lethal unless *secA* is complemented by expression from a plasmid (26). The dependence of *secA* translation on the *secM* CDS would explain the Keio classification as “essential.” However, the IFR within *secM* does not fully correspond with the translation stop sequence, suggesting that there is more to be learned about the translational linkage between the two proteins.

Other researchers have used different approaches to minimize false classification of essential genes during statistical analysis of the insertion profiles by applying a sliding window, quantifying the mean distance between insertions per gene, or variations of truncating the CDS, such as excluding the 3′ end, analyzing only the first 60% of the CDS, or analyzing the central 60% of the CDS (18, 19, 27–31). However, window analysis may overlook genes such as *secM* and analyzing only the first 60% of the CDS would overlook genes such as *ftsK*.

We suggest that the algorithmic approach used here is a more appropriate method for identifying essential chromosomal regions in a sufficiently dense library. However, we see a number of IFRs of >45 bp throughout the genome within nonessential genes, suggesting that our null model of random insertions is not capturing the full structural detail of transposon insertion propensity. This suggests our modeling approach is not based on a perfect representation of biological reality and needs further refinement.

### Polar insertions.

**FIG 4:**
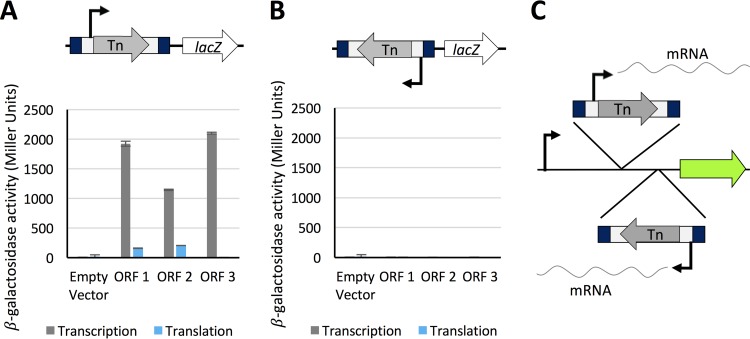
Transcription and translation initiation from within the transposon. The full-length mini-Tn*5* transposon was cloned into expression vectors pRW224 and pRW225 upstream of the *lacZ* gene, in each orientation, for all three open reading frames (ORFs). Vector pRW224 retains a ribosome binding site (RBS) for *lacZ* but no promoter, while vector pRW225 has no promoter or RBS upstream of *lacZ*. Vectors pRW224 and pRW225 can be used to detect transcriptional and translational activity, respectively. β-Galactosidase activity was measured in triplicate for three technical replicates. Values are mean values plus standard deviations between replicates (error bars). (A) Transcriptional read-through was confirmed for one orientation of the transposon, consistent with the orientation of the chloramphenicol gene. Translational read-through from the mini-Tn*5* transposon was confirmed for two out of three open reading frames, consistent with GUG (ORF 1) and AUG (ORF 2) start codons in the transposon inverted repeat. (B) No transcriptional or translational read-through was detected for the opposite orientation of the transposon. (C) Schematic representing the orientation of transposon insertions. The α-orientation of the transposon (top expanded view) corresponds with the chloramphenicol cassette oriented left to right. The β-orientation (bottom expanded view) corresponds with transposon insertions in the opposite direction. An arbitrary gene is represented by the green arrow. The chloramphenicol cassette is denoted by the letters Tn.

A common feature when creating insertion mutants is the introduction of off-target polar effects where expression of adjacent genes is disrupted by the insertion. To mitigate against such polar effects, we designed a cassette that enabled both transcriptional and translational read-through in one direction only. To confirm that transcriptional and translational read-through emanates from the transposon, the transposon was cloned in both orientations and in all three reading frames upstream of the *lacZ* gene in transcription and translation expression vectors pRW224 and pRW225, derivatives of pRW50 (32, 33). Transcriptional read-through was confirmed for one orientation of the transposon, consistent with transcriptional read-through from the chloramphenicol resistance cassette into the downstream disrupted CDS (Fig. 4). Translational read-through was identified for two of the three open reading frames that coincided with AUG and GUG start codons in the inverted repeat at the end of the transposon. More β-galactosidase activity was obtained from the construct in which the AUG codon was in frame than when the GUG codon was in frame, confirming that translation was initiated more strongly from the AUG codon. Therefore, transcription is initiated from within the transposon, and translation is initiated from within the inverted repeat. This allows transcription and translation of downstream essential regions, even from within a CDS. Such events can be identified by determining to which DNA strand the sequencing data maps (Fig. 4C).

Analysis of our data reveals a number of chromosomal regions with insertions in only one orientation. Such insertion profiles can offer insight into transcriptional regulation of genes when considered in conjunction with neighboring genes. For example, the gene *rnc* is located in an operon upstream of the essential gene, *era*. Only mutants with transposons that maintain downstream transcription of *era* are viable (Fig. 3C). Baba et al. categorized *rnc* as essential (1). However, in the case of the Keio library, construction of an *rnc* deletion mutant would disrupt the ability of the native promoter to drive downstream expression of the essential *era* gene, resulting in apparent lethality. Similarly, in both the Keio and PEC databases, *yceQ* is listed as essential, but we observed many insertions in *yceQ*, but in only one orientation (Fig. 3D). The gene is located upstream of the essential gene *rne* and is divergently transcribed. The promoter for *rne* is positioned within *yceQ* (35, 36), and deletion of *yceQ* would remove the promoter for *rne*, resulting in an apparent lethal effect. Our data reveal that while *era* and *rne* are essential, *rnc* and *yceQ* are not essential.

Like *rnc* and *yceQ*, several of the antitoxin genes are reported to be essential in the Keio library but not in our data set or the PEC database (Table S3). Antitoxins are required only if the corresponding toxin gene is functional. One example is *yefM*. We observed a substantial number of insertions in one orientation. Unlike *rnc* and *yceQ* where insertions maintained downstream expression, in the case of *yefM*, the opposite is true; insertions that disrupt expression of the antitoxin but maintain downstream expression of the downstream toxin (*yoeB*) are lethal (Fig. 3E). Scrutiny of our data in this manner reveals that these genes are essential.

Another example of insertion bias is observed in a number of genes at the 3′ end of a transcript, such as *rplI* (Fig. 3F). While *rplI* is not reported as essential, it is worth noting because insertions restricted exclusively to one orientation within the gene cannot be explained by the positional context between an essential gene and promoter. One possible explanation for this observation is that transcription promoted from the transposon produces an antisense RNA that inhibits expression of an essential gene. Insertion bias, irrespective of the underlying cause, can result in false classification of genes when quantifying insertion index scores, as these genes have half as many insertions relative to the rest of the genome. As such, these insertion profiles are to be considered when analyzing data with automated statistical approaches.

### Conditionally essential genes.

In addition to the scenarios listed above, certain genes present challenges for binary classification of essentiality. For example, a gene might code for a protein that is essential at a specific phase of growth, or for growth under certain environmental parameters such as temperature or nutrient availability. Our data reveal a range of these conditionally essential genes. For instance, the Keio and PEC databases list *folK* as essential, whereas we detected multiple insertions within *folK* (Fig. 3G). Loss of *folK* disrupts the ability of the bacterium to produce folate, which is an essential metabolite. However, supplementation of the medium with folate abrogates the requirement for folate biosynthesis. In addition to *folK*, the Keio and PEC databases report *degS* as essential. In our data set, *degS* has a high density of insertions throughout the CDS, suggesting that *degS* is not essential for growth on an agar plate (Fig. 3H). Consistent with this, there is substantial literature showing that *degS* mutants can be isolated, but they either lyse in the stationary phase of growth or rapidly accumulate suppressor mutations (37–40).

The conditional essentiality of such mutants can be tested by growing the transposon library in liquid broth. One would expect that mutants lacking *degS* will lyse and that *folK* mutants will be outcompeted as the limited folate available in the medium is depleted. To test these scenarios, two independent samples of the transposon library were grown in Luria broth (LB) at 37°C for 5 or 6 generations to an optical density at 600 nm (OD_600_) of 1.0 and were then sequenced. These samples, LB1 and LB2, resulted in 5,908,163 and 6,403,324 sequences of which 5,201,711 (88.04%) and 5,382,477 (84.06%), respectively, were mapped to the *E*. *coli* BW25113 genome (Table 1). Insertion index scores were calculated as before (Table S4). As there was a high correlation coefficient of 0.97 between the gene insertion index scores of each technical replicate (Fig. 1C), the data were combined to give a pool of 10,584,188 sequences. Scrutiny of our data revealed substantially fewer *degS* and *folK* mutants after growth in LB, supporting our hypothesis that they are conditionally essential (Fig. 3G and H). Other genes showing similar fitness costs can be identified in the LB outgrowth data set (Table S4).

10.1128/mBio.02096-17.7 TABLE S4  Essential genes identified by TraDIS analysis after outgrowth in LB. Download TABLE S4, XLSX file, 0.3 MB. Copyright © 2018 Goodall et al. 2018 Goodall et al. This content is distributed under the terms of the Creative Commons Attribution 4.0 International license.

### Errors in library construction.

The difficulty in classifying a gene as essential through deletion analysis is the dependence on a negative result to inform classification. Thus, failure to knock out the gene may result in the false classification of a gene as essential. For example, the Keio database originally reported *mlaB* (*yrbB*) as essential. However, our data demonstrate that *mlaB* is nonessential, and this is supported by the literature (41, 42). We have observed similar outcomes for several other genes (Table S3). The reason why knockouts of these genes were not obtained in the construction of the Keio library is unknown.

In addition to the false-positive outcomes described above, we noted several instances of false-negative results within the Keio library database. For example, both our TraDIS data and the PEC database identified 18 genes as essential that are reported as nonessential in the original Keio database (Table S2). Subsequently, Yamamoto et al. (34) demonstrated that for 13 of these mutants, the target gene was duplicated during construction of the Keio library, resulting in a functional protein; these genes are almost certainly essential. Another difficulty that arises when targeting essential genes for mutagenesis is the potential to select for mutants with a compensatory mutation elsewhere in the genome. Our data revealed that *hda* is an essential gene, but it is classified as nonessential in the Keio database. Since the initial description of the Keio library, *hda* has been reported to be essential, but *hda* mutants rapidly accumulate suppressor mutations that restore viability (43–45). We hypothesize that this is an explanation for the observed essentiality of some genes in the TraDIS data set that were described as nonessential by others (Table S3). These effects may arise when creating TraDIS libraries, but the effects are masked by the large number of mutants in the population.

Similarly, in the PEC library, where insertion density is low, essential genes with an insertion in a nonessential region of the gene will be falsely classified as nonessential when relying on single insertion mutants to inform essentiality. An example of this false-negative classification in the PEC database is *tadA* (Table S3). The TadA protein is a tRNA-specific deaminase, and its essentiality is reported in the Keio database and our data set and is supported by the literature (46). The PEC database reports a single insertion site within the extreme 3′ end of the *tadA* gene.

We have identified a range of underlying causes behind data set discrepancies and highlight that there are numerous possible insertion profiles for an “essential” gene. As such, it is important to note that no single statistical method, to our knowledge, would fully identify every essential gene and that manual inspection of data is crucial.

### Genes identified as essential only by TraDIS.

**FIG 5:**
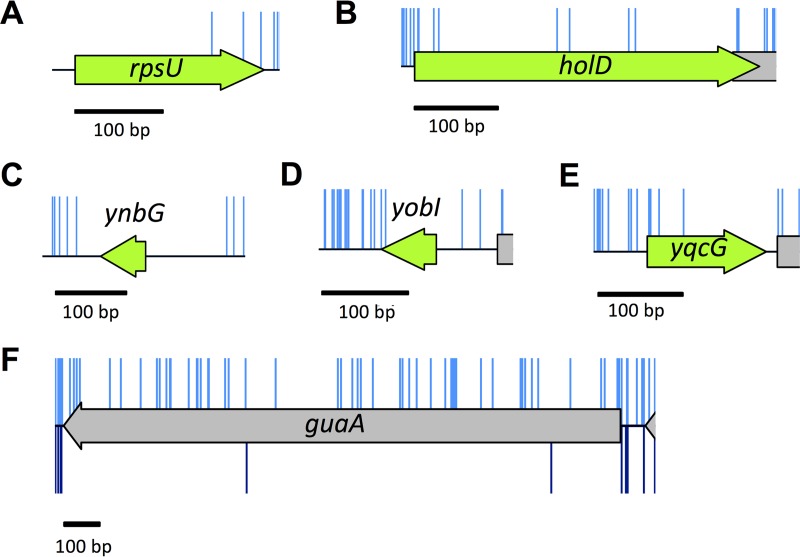
Essential genes unique to our data. (A to E) There are very few or no insertions within these genes in our input library (blue). (A and B) Low insertion frequency and literature support classification of these genes as essential. (C to E) Recently annotated genes with few or no insertions. Our data suggest that these genes are potentially essential or important for growth. (F) The *guaA* gene has a sufficiently low insertion index score to be classified as essential after initial statistical analysis (shown in blue above the schematic representation). Following outgrowth, there are few *guaA* mutants (shown in dark blue below the schematic representation), consistent with literature reports that *guaA* mutants have a growth defect.

There are 81 genes identified as essential using our insertion index data, which are not reported as essential in the Keio or PEC database (Table 2). These genes fall into two groups, those with no insertions and the remainder with insertions in the CDS. The first group is most likely to be essential. For example, *rpsU* is essential in our data and has been described as essential by others (Fig. 5A) (47). However, in the Keio library, there is a duplication event, which gives rise to a mutant that produces a functional protein (34).

**TABLE 2:** Genes with low insertion index scores identified by TraDIS only

Gene group and gene	Function^a^	Comment
Genes with no insertions		
* glyA*	Serine hydroxymethyltransferase	
* pheM*	*pheST* operon leader peptide	
* rimM*	Ribosome maturation protein	
* rplK*	50S ribosomal subunit	Duplicated in Keio collection^b^
* rplY*	50S ribosomal subunit	Duplicated in Keio collection
* rpmF*	50S ribosomal subunit	
* rpmI*	50S ribosomal subunit	
* rpsF*	30S ribosomal subunit	
* rpsO*	30S ribosomal subunit	Duplicated in Keio collection
* rpsT*	30S ribosomal subunit	
* rpsU*	30S ribosomal subunit	Duplicated in Keio collection
* thyA*	Thymidylate synthase	
* ttcC*	Pseudogene	
* ynbG*	Small protein	

Genes with a low frequency of insertions		
* aceF*	Pyruvate dehydrogenase, E2 subunit	
* cydB*	Cytochrome *bd*-I terminal oxidase subunit II	
* cydD*	Glutathione/l-cysteine exporter	
* cydX*	Cytochrome *bd*-I terminal oxidase	
* dapF*	Diaminopimelate epimerase	
* dcd*	dCTP deaminase	
* fabH*	β-Ketoacyl-ACP synthase III	
* fdx*	Reduced ferredoxin	
* folP*	Dihydropteroate synthase	Duplicated in Keio collection
* guaA*	GMP synthetase	
* hemE*	Uroporphyrinogen decarboxylase	Duplicated in Keio collection
* higA*	Antitoxin of the HigB-HigA toxin-antitoxin system	
* hipB*	HipB antitoxin and DNA-binding transcriptional repressor	
* holD*	DNA polymerase III, Ψ subunit	
* hscA*	Chaperone for [Fe-S] cluster biosynthesis	
* ihfA*	Integration host factor (IHF), α subunit	
* iraM*	Antiadaptor protein IraM, inhibitor of σ^S^ proteolysis	
* iscS*	Cysteine desulfurase	
* iscU*	Scaffold protein for iron-sulfur cluster assembly	
* lipA*	Lipoyl synthase	
* lpd*	Lipoamide dehydrogenase	
* lpxL*	Lauroyl acyltransferase	
* lysS*	Lysine-tRNA ligase	
* mnmA*	tRNA-specific 2-thiouridylase	
* pdxH*	Pyridoxine 5′-phosphate oxidase/pyridoxamine 5′-phosphate oxidase	
* priB*	Primosomal replication protein N	Duplicated in Keio collection
* ptsI*	PTS enzyme I	
* rbfA*	30S ribosome-binding factor	
* relB*	RelB antitoxin and DNA-binding transcriptional repressor	
* rluD*	23S rRNA pseudouridine synthase	
* rnt*	RNase T	
* rpe*	Ribulose-5-phosphate 3-epimerase	
* rplA*	50S ribosomal subunit	
* safA*	EvgS/EvgA and PhoQ/PhoP connector	
* sucA*	2-Oxoglutarate decarboxylase	
* sucB*	Dihidrolipoyltransuccinylase	
* tktA*	Transketolase I	
* tonB*	TonB energy transducing system, TonB subunit	
* trpL*	*trp* operon leader peptide	
* tusE*	Sulfur transfer protein	
* ubiE*	Bifunctional 2-octaprenyl-6-methoxy-1,4-benzoquinone methylase and *S*-adenosylmethionine:2-DMK methyltransferase	
* ubiG*	Bifunctional 3-demethylubiquinone-8 3-*O*-methyltransferase and 2-octaprenyl-6-hydroxyphenol methylase	
* ubiH*	2-Octaprenyl-6-methoxyphenol hydroxylase	
* ubiX*	3-Octaprenyl-4-hydroxybenzoate carboxy-lyase partner protein	
* ybeY*	Endoribonuclease involved in maturation of 16S rRNA and ribosome quality control	
* ycaR*	Conserved protein	
* yciS*	Lipopolysaccharide assembly protein	
* ydaE*	Rac prophage; zinc-binding protein	
* ydaS*	Rac prophage; predicted DNA-binding transcriptional regulator	
* ydcD*	Hypothetical protein	
* yddL*	Predicted lipoprotein	
* ydfO*	Qin prophage; predicted protein	
* ydhL*	Conserved protein	
* yedN*	Putative protein	
* yffS*	CPZ-55 prophage	
* ygeF*	Predicted protein	
* ygeG*	Predicted chaperone	
* ygeN*	Predicted protein	
* ygfZ*	Folate-binding protein	
* yjbS*	Hypothetical protein	
* ykfM*	Hypothetical protein	
* ymfE*	e14 prophage; predicted inner membrane protein	
* ymiB*	Putative protein	
* yncH*	Hypothetical protein	
* yobI*	Small protein	
* yqcG*	Cell envelope stress response protein	
* yqeL*	Small protein	

a ACP, acyl carrier protein; PTS, phosphotransferase system; DMK, demethylmenaquinone. Functions are taken from the Ecocyc website.

b Yamamoto et al. (34).

Scrutiny of our data for the remaining genes reveals that there are additional essential genes with a low frequency of insertions. For instance, *holD* has been described in the literature as an essential gene (48). Our data support that finding (Fig. 5B and Table S1). However, *holD* mutants are available in the Keio collection. The demonstration by Durand et al. and others (48–50) that *holD* mutants accumulate extragenic suppressor mutations at high frequency may explain why these mutants are considered nonessential in the Keio database and why we observe a low frequency of insertions in our experiments.

A number of the genes unique to our analysis were not identified as essential in the Keio collection or PEC database simply because they are not included in either of these data sets. This is in part because the Keio collection of knockout mutants was based on available annotation data at the time (51). For example, the identification and location of *ynbG*, *yobI*, and *yqcG* were published only in 2008 (52). These genes show very sparse or no transposon disruption in our data, and consequently, these genes are potentially essential (Fig. 5C, D, and E). Further validation studies would be required to confirm this.

As mentioned previously, overreporting of essential genes may occur when nonessential genes have low insertion index scores. Such low insertion index scores may arise due to attenuated growth. An example of gene misclassification because mutation results in a fitness cost and attenuated growth is *guaA*. The low insertion index score results in *guaA* being classed as essential despite having many insertions. The fitness effect was confirmed by growing the library in LB, as such mutants are outcompeted (Fig. 5F), and the literature supports the fact that this gene is not essential and has an altered growth rate (53).

### High-resolution features within a TraDIS data set.

**FIG 6:**
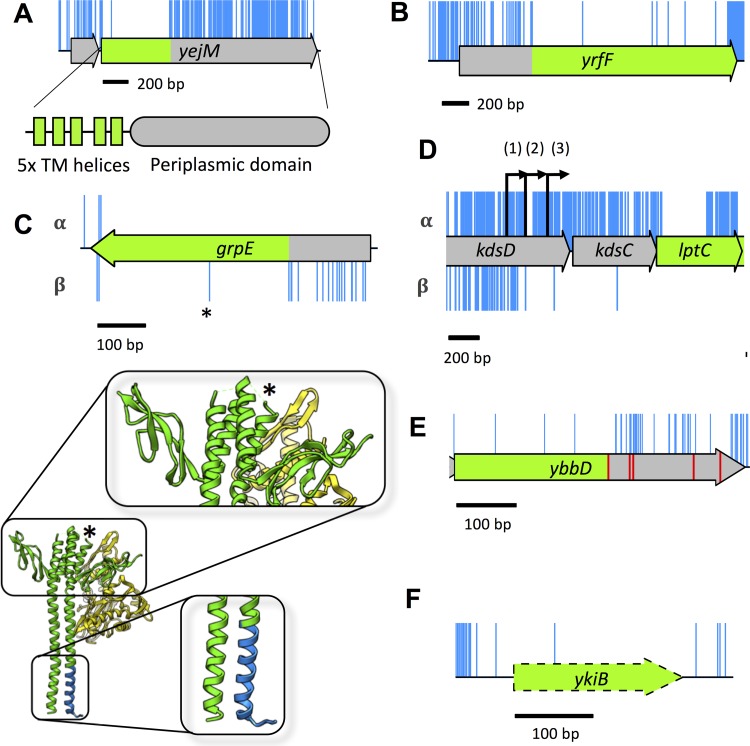
Additional features identified through detailed analysis of high-resolution insertion data. (A) Insertions within the *yejM* CDS, but not along the full length, correspond with a nonessential periplasmic domain. The 5′ end of the CDS has no insertions and corresponds with five essential transmembrane (TM) domains of YejM. (B) Insertions within *yrfF* suggest a dispensable 5′ domain. (C) The *grpE* gene tolerates transposon insertions in the 5′ end of the CDS (blue), but only in the orientation that maintains expression of the downstream protein (lower track, β-orientation). The GrpE protein forms a dimer (green) which interacts with DnaK (yellow). Transposon insertions in specific regions of the protein do not disrupt GrpE interaction with DnaK (blue). An additional, single, insertion point in the center of the *grpE* CDS (indicated by an asterisk) maps back to a turn between two helices of the GrpE protein. The data reveal dispensable sections of the GrpE protein and boundaries in secondary structure. (D) Insertions immediately upstream of *lptC* have an insertion orientation bias. Only insertions that maintain expression of *lptC* are tolerated within *kdsC* (α-orientation). The *lptC* gene has three promoters (indicated by the numbers 1, 2, and 3 within parentheses above the black arrows); the insertion boundary indicate that promoter 2 is the essential promoter. (E) Pseudogene *ybbD* contains many more insertions after the first stop codon (red), suggesting that the truncated CDS may still be functional and essential. (F) The pseudogene *ykiB* is not annotated in the BW25113 genome (CP009273.1) and has a single insertion within the CDS.

Manual inspection of a TraDIS data set can reveal additional information that might go unnoticed in a high-throughput analysis pipeline. A common observation from this and previous detailed analysis of data from saturated transposon libraries is the ability to determine, at the base pair level of resolution, the boundaries of essential regions within a gene. An example of an essential gene with a dispensable 3′ end is *yejM* (*pbgA* in *Salmonella enterica* serotype Typhimurium). Only the 5′ end of the CDS is essential, up to and including codon 189, which corresponds with five transmembrane helices of the protein structure; the C terminus of the protein is a periplasmic domain that is dispensable for viability (Fig. 6A) (54–56). Our TraDIS data revealed insertions in codons 186 and 189. Analysis of the transposon orientation at these points revealed that they corresponded with the same transposon insertion location but, due to the 9-bp duplication introduced by the transposon, in different transposon orientations. The introduced transposon sequence maintains codon 189, completely consistent with previously reported results (54, 56).

In addition, as a result of our transposon design, a further feature of our TraDIS data is the identification of genes with dispensable 5′ ends. An example of this is *yrfF*, which encodes an inhibitor of the Rcs stress response (Fig. 6B) (57, 58). This phenomenon, while less well covered in the literature, is not surprising, given that Zhang et al. report equal likelihood of a required intragenic region residing at the 5′ or 3′ end of a gene, albeit in *Mycobacterium tuberculosis* (31). These mutants will be viable only if the remaining CDS can be translated into a functional product, and one would expect to find an orientation bias where the transposon drives downstream transcription and translation of the essential region.

Interestingly, inspection of our data revealed essential genes with isolated insertions within the coding sequence. An example of this is *grpE*. The *grpE* gene codes for the essential nucleotide exchange factor that forms a dimer and interacts with the DnaK/J complex (59). The isolated insertion occurs only in the orientation that maintains expression of the remaining CDS (Fig. 6C). Mapping of the site of transposon insertion onto the previously determined protein structure of GrpE indicated that the insertion occurred within the part of the gene encoding a flexible linker between two α-helices (Fig. 6C). This observation suggests that similar to the GAL4 protein of *Saccharomyces cerevisiae* used in the yeast two-hybrid system (60), the adenylate cyclase from *Bordetella pertussis* used in the bacterial two-hybrid system (61), and split green fluorescent protein (GFP) (GFP1-10 and GFP11) (62), a functional GrpE can be expressed as two separate essential domains that interact to form a functional protein. Therefore, the data presented here can be exploited to identify every essential gene that could be used in a protein fragment complementation assay to develop similar protein-protein interaction screens. Importantly, our TraDIS library has an unprecedented sub-CDS level of resolution that can demarcate changes in the protein secondary structure.

Another fine-resolution mapping feature of our transposon data is the identification of the promoter position for essential genes, as also previously reported by Christen et al. (8). An example of this is the promoter for *lptC*, located within the *kdsD* gene. Three promoters [kdsCp3 (1), kdsCp2 (2), kdsCp1 (3)] have been identified within the *kdsD* gene (63, 64). However, our data show that insertions that maintain expression of *lptC* are tolerated within the *kdsD* gene up to kdsCp2; insertions stop short just before the kdsCp2 −35 consensus sequence, excluding a single insertion between the −35 and −10 positions, and a single insertion further downstream (Fig. 6D). These results suggest that kdsCp3 is dispensable and that at least kdsCp2 is required for adequate expression of *lptC*. As in the case of *grpE* above, this is another example of the unprecedented level of genetic detail that can be obtained from this high-throughput method.

Finally, within our data we observed a number of transposon-free sections that do not correspond with the annotated features of our genome. This can occur when a start codon is misannotated (8), or translation might initiate at a secondary start codon downstream of the transposon insertions. Alternatively, a pseudogene annotation may extend beyond the first stop codon. One example of this is *ybbD*, a pseudogene classified as nonessential in our data set. However, the annotation of *ybbD* in the BW25113 genome extends beyond the first stop codon, whereas in other genomes it does not. Our data find that the region from the methionine translation start codon to the first stop codon passes the threshold for essentiality (Fig. 6E). In addition, we observed a transposon-free region corresponding to *ykiB* (Fig. 6F). This gene is annotated in *E*. *coli* W3110 but not in BW25113, despite the fact the nucleotide sequence is identical. Our data suggest that these genes have a significant role in viability or growth, but further investigation is required to test this hypothesis. These examples highlight the importance of having a fully annotated and curated reference genome for mapping data. However, even the highly studied K-12 genome with its wealth of annotation information retains some as yet unexplained transposon-free regions. Thus, TraDIS can help identify regions of genomes where annotation is incorrect or incomplete.

### Conclusion.

In summary, comparison of the TraDIS data with data from two previous studies of *E. coli* K-12 under standard laboratory conditions revealed 248 genes designated essential in all three data sets (Table S2). We have shown why different conclusions have been drawn from transposon mutagenesis data and gene deletion studies. Essential genes that contain both essential and nonessential regions will statistically appear nonessential if judged only on insertion index scores. We have demonstrated the importance of visual analysis to avoid automation bias in designating genes as essential or nonessential. We have also identified genes incorrectly designated essential because of the failure to recognize polarity effects on a downstream essential gene in the same transcription unit. We also report potential new essential genes and discuss the use of transposon sequencing for fine-resolution mapping of features across the genome. Importantly, TraDIS data are a valuable resource that can be reinspected following the discovery of new features within a given genome. Finally, our data reveal that there is more to be understood about genome structure and organization, which further coupling of modeling and experimental approaches will help to elucidate.

## MATERIALS AND METHODS

### Strains and plasmids.

*E. coli* K-12 strain BW25113, the parent strain of the Keio library, was used for construction of a transposon library. The strain has the following genotype: *rrnB3* Δ*lacZ4787 hsdR514* Δ(*araBAD*)*567* Δ(*rhaBAD*)*568 rph-1* (65). The transposon mutant library was constructed by collaborators from Discuva Ltd., Cambridge, United Kingdom, following a method described for *Salmonella* Typhi (4). The main differences were that a mini-Tn*5* transposon coding for a chloramphenicol resistance cassette was used. This was amplified by PCR from the *cat* gene of the plasmid vector pACYC184 (66) using oligonucleotide primers incorporating the Tn*5* transposon mosaic ends. Transposomes were prepared using Tn*5* transposase (Epicentre, Madison, WI, USA), and these were introduced into *E. coli* K-12 strain BW25113 by electrotransformation. Transposon mutants were selected by growth on LB agar supplemented with chloramphenicol. Approximately 5.6 million colonies representing an estimated 3.7 million mutants were pooled and stored in 15% glycerol at −80°C.

### Media and growth conditions.

DNA was extracted from two samples of the transposon library glycerol stock to generate TraDIS data referred to as TL1 and TL2 in the text. In addition, DNA was extracted from two independent cultures, LB1 and LB2, of the library grown in Luria broth (LB) (10 g tryptone, 5 g yeast extract, 10 g NaCl) and grown for generations at 37°C with shaking until the culture reached an optical density at 600 nm (OD_600_) of 1.0.

### β-Galactosidase assay.

β-Galactosidase assays were used to measure the activity of transposon::*lacZ* fusions. The transposon was cloned in each orientation, for all three open reading frames, into transcription and translation assay vectors pRW224 and pRW225 (33). Strains carrying the transposon::*lacZ* fusions were grown overnight at 37°C with aeration in LB supplemented with 35 μg/ml tetracycline (Sigma). The density of the overnight culture was determined by measuring OD_650_ and then used to subculture into 5 ml LB and incubated at 37°C with aeration until the mid-exponential phase of growth (OD_650_ of 0.3 to 0.5). Each culture was lysed by adding 100 μl each of toluene and 1% sodium deoxycholate, mixed by vortexing for 15 s and aerating for 20 min at 37°C. The β-galactosidase activity of each culture was assayed by the addition of 100 μl of each culture lysate for three technical replicates to 2.5 ml Z buffer (10 mM KCl, 1 mM MgSO_4_ ⋅ 7H_2_O, 60 mM Na_2_HPO_4_, 30 mM NaH_2_PO_4_ ⋅ 2H_2_O supplemented with 2.7 ml β-mercaptoethanol per liter of distilled water, adjusted to pH 7) supplemented with 13 mM 2-nitrophenyl-β-d-galactopyranoside (ONPG) (Sigma). The reaction mixture was incubated at 37°C until a yellow color had developed, after which the reaction was stopped by adding 1 ml of 1 M sodium carbonate. The absorbance of the reaction at OD_420_ was measured, and β-galactosidase activity was calculated in Miller units.

### TraDIS sequencing.

Harvested cells were prepared for sequencing following an amended TraDIS protocol (4, 8, 9). Genomic DNA was isolated using a Qiagen QIAamp DNA blood minikit, according to the manufacturer’s specifications. DNA was quantified and mechanically sheared by ultrasonication. Sheared DNA fragments were processed for sequencing using NEB Next Ultra I kit. Following adaptor ligation, a PCR step was introduced to enrich for transposon-containing fragments, using a forward primer specific for the transposon 3′ end and a reverse primer specific for the adaptor. After PCR purification, an additional PCR prepared DNA for sequencing through the addition of Illumina-specific flow cell adaptor sequences and custom inline index barcodes of variable length in the forward primers. The purpose of this was to increase indexing capacity while staggering introduction of the transposon sequence to increase base diversity during sequencing. Samples were sequenced using Illumina MiSeq 150 cycle v3 cartridges, aiming for an optimal cluster density of 800 clusters per mm^2^.

### Sequencing analysis.

Raw data were collected and analyzed using a series of custom scripts. The Fastx barcode splitter and trimmer tools, of the Fastx toolkit, were used to assess and trim the sequences (67). Sequence reads were first filtered by their inline indexes, allowing no mismatches. Transposon similarity matching was done by identifying the first 35 bp of the sequenced transposon in two parts: 25 bases (5′ to 3′, corresponding to the PCR2 primer binding site) were matched, allowing for three mismatches, trimmed, and then the remaining 10 bases (corresponding to the sequenced transposon) matched, allowing for one mismatch, and trimmed. Sequences less than 20 bases long were removed using Trimmomatic (68). Trimmed, filtered sequences were then aligned to the reference genome *E. coli* BW25113 (accession no. CP009273.1), obtained from the NCBI genome repository (69). Where gene names differed between databases, the BW25113 annotation was used. The aligner bwa was used, with the mem algorithm (0.7.8-r455 [75]). Aligned reads were filtered to remove any soft clipped reads. The subsequent steps of conversion from SAM (sequence alignment/map) files to BAM (binary version of SAM) files, and the requisite sorting and indexing, were done using SAMtools (0.1.19-44428cd [70]). The BEDTools suite was used to create BED (browser extensible data) files which were intersected against the coding sequence boundaries defined in general feature format (.gff) files obtained from the NCBI (71). Custom python scripts were used to quantify insertion sites within the annotated CDS boundaries. Data were inspected manually using the Artemis genome browser (72).

### Essential gene prediction.

The frequency of insertion index scores was plotted in a histogram using the Freedman-Diaconis rule for choice of bin widths (see Fig. S1 in the supplemental material). Using the R MASS library (http://www.r-project.org), an exponential distribution (red line) was fitted to the left, “essential” mode (i.e., any data to the left of the trough in Fig. S1); a gamma distribution (blue line) was fitted to the right, “nonessential” mode (i.e., any data to the right of the trough). The probability of a gene belonging to each mode was calculated, and the ratio of these values was used to calculate a log likelihood score. Using a 12-fold likelihood threshold, based on the log likelihood scores, genes were assigned as “essential” if they were 12 times more likely to be in the left mode than in the right mode, and “nonessential” if they were 12 times more likely to be in the right mode (9). Genes with log likelihood scores between the upper and lower log_2_ 12 threshold values of 3.6 and −3.6, respectively, were deemed “unclear.” A threshold cutoff of log_2_(12) was chosen, as it is more stringent than log_2_(4) (used by Langridge et al. [4]), and consistent with analysis used by Phan et al. (9).

### Essential gene lists.

The Keio essential gene list is composed of the original essential genes minus three open reading frames (ORFs), JW5190, JW5193, and JW5379, as they are not annotated within strain MG1655 and are thought to be spurious, giving a final list of 300 genes (1, 73). The PEC data set is composed of the 300 genes listed as essential for strain W3110 (2). The lists of essential genes were compared using BioVenn (74).

### Statistical analysis.

For details of the statistical analysis, see Text S1, Fig. S1, and Fig. S2 in the supplemental material.

### Accession number(s).

TraDIS sequencing data are available from the European Nucleotide Archive under accession no. PRJEB24436.
