# Supplementary figures and images for: The Essential Genome of Escherichia coli K-12
Source: mBio. 2018 Feb 20;9(1):e02096-17. doi: 10.1128/mBio.02096-17 (PMC5821084; doi:10.1128/mBio.02096-17)

**A**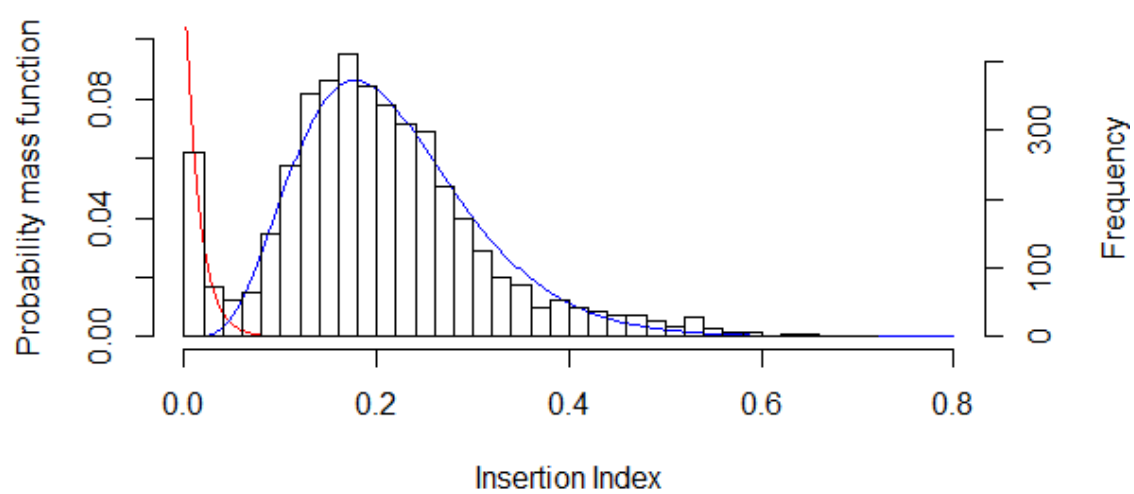**B**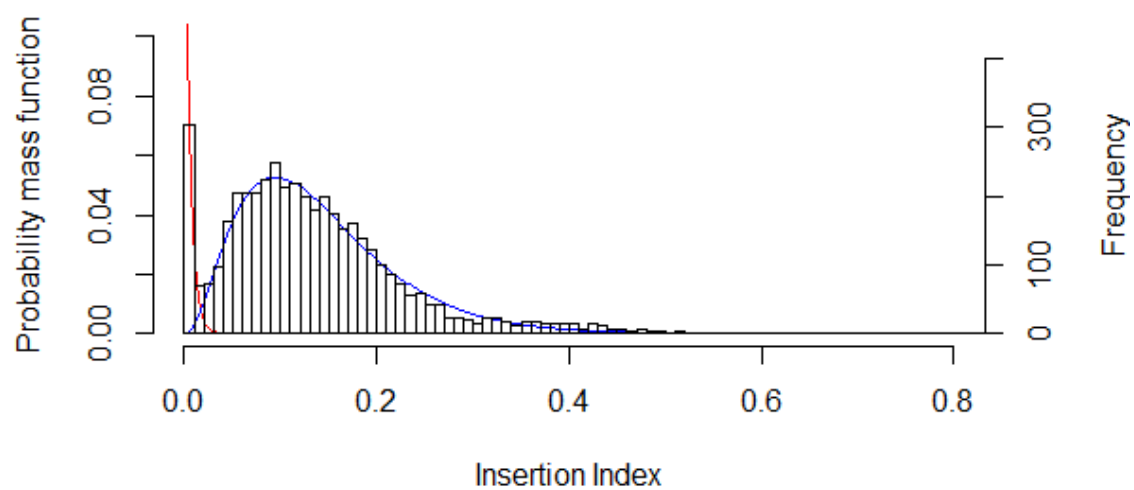

Supplement: FIG S1 [file mbo001183726sf1.pdf]

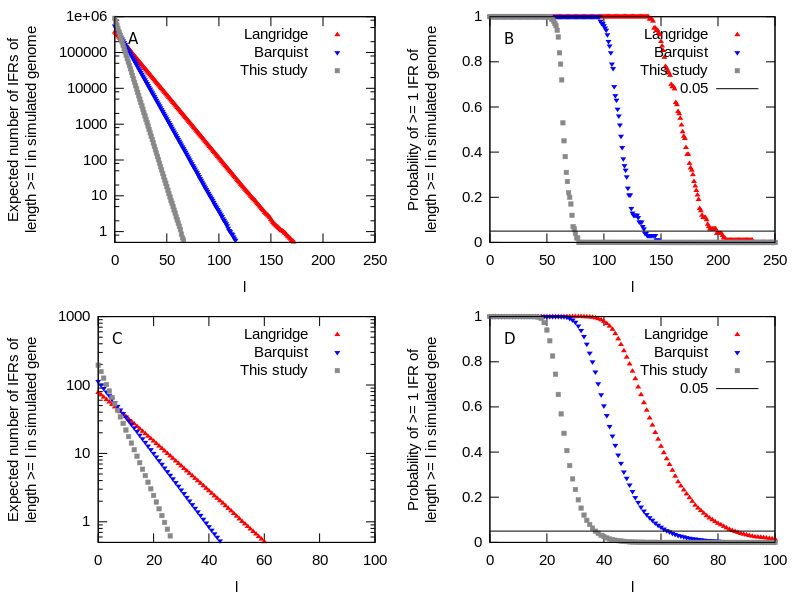

Supplement: FIG S2 [file mbo001183726sf2.tif]
