# Supplementary material for: The Essential Genome of Escherichia coli K-12
Source: mBio. 2018 Feb 20;9(1):e02096-17. doi: 10.1128/mBio.02096-17 (PMC5821084; doi:10.1128/mBio.02096-17)
Supplement: TABLE S2 [file mbo001183726st2.pdf]

Table S2. Comparison of essential genes identified by Keio, PEC and TraDIS

TraDIS only	Keio only	PEC only (W3110)	TraDIS-Keio	TraDIS-PEC	Keio-PEC	All 3
aceF	*alsK*	*argU*	*cydA*	*alaS*	*degS*	*lptD (imp)*
cydB	*bcsB*	*argX*	*cydC*	*coaA*	*folK*	*erpA (yadR)*
cydD	*mazE (chpR)*	*cysT*	*dicA*	*coaE*	*ftsE*	*bamA (yaeT)*
cydX (ybgT) [a]	*chpS*	*efp*	*purB*	*dnaG*	*ftsK*	*lptE (rlpB)*
dapF	*entD*	*ffs*	*racR*	*dnaT*	*ftsN*	*murJ (mviN)*
dcd	*minD*	*glyT*	*rpoE*	*folB*	*ftsX*	*prs (prsA)*
fabH	*minE*	*hisR*	*tadA*	*glmM*	*ribB*	*tsaB (yeaZ)*
fdx	*waaU (rfaK)*	*kdsC*	*ubiB*	*glyS*	*rne*	*bamD (yfiO)*
folP	*rnc*	*leuU*	*ubiD*	*groL*	*secD*	*nadK (yfjB)*
glyA	*tdcF*	*leuW*	*wzyE*	*hda*	*secF*	*tsaD (ygjD)*
guaA	*tnaB*	*leuZ*	*cohE (ymfK)*	*ileS*	*secM*	*lptA (yhbN)*
hemE	***yabQ*** [bcd]	*polA*		*nusB*	*spoT*	*tsaC (yrdC)*
higA	*yafF* [c]	*priA*		*parC*	*rseP (yaeL)*	*waaA (kdtA)*
hipB	*yagG*	*proM*		*prfB*	*yceQ*	*tsaE (yjeE)*
holD	*ydfB*	*serT*		*rho*	*yejM*	*lptF (yjgP)*
hscA	*ydiL*	*serV*		*rpoD*	*lptC (yrbK)*	*lptG (yjgQ)*
ihfA	*yefM*	*thrU*		*rsgA*		*ribF*
iraM	*mqsA (ygiT)*	*trpT*		*lptB (yhbG)*		*lspA*
iscS	*yhbV*					*ispH*
iscU	*yhhQ*					*dapB*
lipA	***yibJ*** [bce]					*folA*
lpd	*ubiJ (yigP)*					*ftsL*
lpxL	***yqgD*** [bf]					*ftsI*
lysS	*rsmI (yraL)*					*murE*
mnmA	*mlaB (yrbB)*					*murF*
pdxH						*mraY*
pheM						*murD*
priB						*ftsW*
ptsI						*murG*
rbfA						*murC*
relB						*ftsQ*
rimM						*ftsA*
rluD						*ftsZ*
rnt						*lpxC*
rpe						*secA*
rplA						*can*
rplK						*hemL*
rplY						*dapD*
rpmF						*map*
rpmI						*rpsB*
rpsF						*tsf*

rpsO
rpsT
rpsU
safA
sucA
sucB
thyA
tktA
tonB
trpL
ttcC
tusE (yccK)
ubiE
ubiG
ubiH
ubiX
ybeY
ycaR
yciS
ydaE
ydaS
ydcD
yddL
ydfO
ydhL
yedN
yffS
ygeF
ygeG
ygeN
ygfZ
yjbS
ykfM
ymfE
ymiB
ynbG
yncH
yobI
yqcG
yqeL

pyrH
frr
dxr
ispU
cdsA
lpxD
fabZ
lpxA
lpxB
dnaE
accA
tilS
proS
hemB
ribD
ribE
thiL
dxs
ispA
dnaX
adk
hemH
lpxH
cysS
folD
mrdB
mrdA
nadD
holA
leuS
lnt
glnS
fldA
infA
lolA
serS
rpsA
msbA
lpxK
kdsB
mukF
mukE
mukB
asnS
fabA

fabD
fabG
acpP
tmk
holB
lolC
lolD
lolE
pth
ispE
lolB
hemA
prfA
prmC
kdsA
topA
ribA
fabI
tyrS
ribC
pheT
pheS
rplT
infC
thrS
nadE
gapA
aspS
argS
pgsA
metG
folE
gyrA
nrdA
nrdB
folC
accD
fabB
gltX
ligA
zipA
dapE
dapA
der
hisS

ispG
suhB
acpS
era
lepB
pssA
rplS
trmD
rpsP
ffh
grpE
csrA
ispF
ispD
ftsB
eno
pyrG
lgt
fbaA
pgk
metK
yqgF
plsC
parE
cca
infB
nusA
ftsH
obgE
rpmA
rplU
ispB
murA
rpsI
rplM
mreD
mreC
mreB
accB
accC
def
fmt
rplQ
rpoA
rpsD

rpsK
rpsM
secY
rplO
rpmD
rpsE
rplR
rplF
rpsH
rpsN
rplE
rplX
rplN
rpsQ
rpmC
rplP
rpsC
rplV
rpsS
rplB
rplW
rplD
rplC
rpsJ
fusA
rpsG
rpsL
trpS
yrfF
asd
rpoH
ftsY
glyQ
gpsA
coaD
rpmB
dfp
dut
gmk
gyrB
dnaN
dnaA
rpmH
rnpA
yidC

glmS
glmU
hemD
hemC
hemG
yihA
murI
murB
birA
secE
nusG
rplJ
rplL
rpoB
rpoC
ubiA
plsB
lexA
dnaB
ssb
groS
psd
orn
rpsR
ppa
valS
dnaC

[a]BW25113 (CP009273.1) gene names have been used unless otherwise specified. Alternative gene names used by Keio or PEC are shown in brackets

[b]The Keio naming convention is used for all genes in bold

[c]The annotation of this gene varies between the Keio collection and BW25113 (CP009273.1)

[d]*yabQ* is not annotated in BW25113 (CP009273.1), but corresponds with the 2nd half of *yabP*

[e]*yibJ* is annotated as *rhsJ* in BW25113 (CP009273.1), but the annotation of *rhsJ* extends beyond the first stop codon

[f]*yqgD* is not annotated in BW25113 (CP009273.1)
