# Supplementary material for: The Essential Genome of Escherichia coli K-12
Source: mBio. 2018 Feb 20;9(1):e02096-17. doi: 10.1128/mBio.02096-17 (PMC5821084; doi:10.1128/mBio.02096-17)
Supplement: TEXT S1 [file mbo001183726s1.docx]

**Supplementary Methods**

**Statistical Analysis**

A Poissonian model, parameterized only by insertion density, gives the probability that no insertions will be found in a single region of a given length (for example, 60 bases), whereas genes and genomes have many such regions that are effectively independent. Briefly, if a p-value of 0.05 is associated with an IFR of length 60 under a simple Poisson model, every length-60 region in the gene or genome of interest has around a 5% chance of being insertion-free by chance under the null hypothesis. As a genome will contain many thousand such regions, many IFRs of length 60 will be expected by chance under the null hypothesis. A simulation of random insertion events reveals ~2900 IFRs of length 60 or above by random chance in a 4.8Mb genome with 370, 000 insertions (red markers in Fig. S1A).

To correct for this, we need to carefully state the statistic of interest and the corresponding null model. At least three probabilities are pertinent here: those with which, under a null model of random, independent insertions, (i) a single length-l region has no insertions; (ii) a gene of length g contains one or more IFRs of length l; (iii) a genome of length G contains one or more IFRs of length l. Previous models have implicitly estimated only (i), but to control the false discovery rate for individual genes or on a genome-wide basis, (ii) and (iii) respectively, are required. An analytic calculation of the corrected p-values corresponding to (ii) and (iii), which we label p_gene and p_genome, is laborious; for simplicity and illustrative power we computationally investigate these statistics, simulating many instances of N random non-coincident insertions in a genome of length G and reporting the statistics of resultant IFRs (C code available as SI). Reinterpreting the statistics in previous papers on this topic, and assuming a representative gene length of g = 1000, we find (Fig. S1) that in Langridge *et al.* (371,775 inserts, 4,791,961 base genome), the null model of genome-wide random insertion gives an expected ~16,000 IFRs with l >= 39 (classified in the original study using the Poisson model as p < 0.05) and ~2,900 IFRs with l >= 60 (classified as p < 0.01); the corrected lengths for p_genome = 0.05 and p_gene = 0.05 are ~200 and ~85 respectively (Figures S1B,D; [1]). In Barquist *et al.* (549,086 inserts, 4,878,012 base genome), the null model of genome-wide random insertion gives an expected ~22,000 IFRs of l >= 27 (classified as p < 0.05) and ~4,100 IFRs of l >= 41 (classified as p < 0.01); the corrected lengths for p_genome = 0.05 and p_gene = 0.05 are ~135 and ~62 respectively [2]. Our study (901,383 inserts, 4,631,469 base genome) gives a corrected p_genome = 0.05 of ~75 (and p_gene = 0.05 of ~36).

A further complication arises because the probability of observing an IFR of given length in a gene and the labelling of that gene as essential are not trivially related. Rather, genes are classified as essential if their insertion score clusters in the low-score category. While the analysis above can therefore give an indication of the resolution of our experiment, further theory, perhaps grounded in Hidden Markov Model analysis [3], will be required to forge a rigorous connection between the statistics of IFRs and the statistical power associated with classification of genes.

[1] Langridge GC, Phan MD, Turner DJ, Perkins TT, Parts L, Haase J, Charles I, Maskell DJ, Peters SE, Dougan G, Wain J, Parkhill J, Turner AK. 2009. Genome Res 19:2308–2316.

[2] Barquist L, Langridge G, Turner D, Phan M, Turner AK, Bateman A, Parkhill J, Wain J, Gardner P. 2013. Nucleic Acids Res 41:4549-4564.

[3] DeJesus MA, Iorger TR. 2013. BMC Bioinformatics 14:303.
