# Supplementary material for: The Essential Genome of Escherichia coli K-12
Source: mBio. 2018 Feb 20;9(1):e02096-17. doi: 10.1128/mBio.02096-17 (PMC5821084; doi:10.1128/mBio.02096-17)
Supplement: TABLE S3 [file mbo001183726st3.pdf]

Table S3. Causes of discrepancies between datasets

Gene	Venn group	Cause of discrepancy
alsK	K	Errors in library construction
bcsB	K	Errors in library construction
chpS	K	Anti-Toxin
entD	K	Errors in library construction
mazE (chpR)	K	Anti-Toxin
minD	K	Errors in library construction
minE	K	Errors in library construction
mlaB (yrbB)	K	Errors in library construction
mqsA (ygiT)	K	Genes containing a transposon free region
rnc	K	Polar insertions
rsmI (yraL)	K	Errors in library construction
tdcF	K	Unclear
tnaB	K	Errors in library construction
ubiJ (yigP)	K	Polar insertions
waaU (rfaK)	K	Genes containing a transposon free region
yabQ	K	Genes containing a transposon free region
yafF	K	Genes containing a transposon free region
yagG	K	Unclear
ydfB	K	Unclear
ydiL	K	Unclear
yefM	K	Anti-Toxin
yhbV	K	Unclear
yhhQ	K	Errors in library construction
yibJ	K	Genes containing a transposon free region
yqgD	K	Genes containing a transposon free region
degS	KP	Conditionally essential
folK	KP	Conditionally essential
ftsE	KP	Errors in library construction
ftsK	KP	Genes containing a transposon free region
ftsN	KP	Genes containing a transposon free region
ftsX	KP	Genes containing a transposon free region
lptC (yrbK)	KP	Genes containing a transposon free region
ribB	KP	Genes containing a transposon free region
rne	KP	Genes containing a transposon free region
rseP (yaeL)	KP	Conditionally essential
secD	KP	Errors in library construction
secF	KP	Errors in library construction
secM	KP	Genes containing a transposon free region
spoT	KP	Genes containing a transposon free region
yceQ	KP	Polar insertions
yejM	KP	Genes containing a transposon free region
cohE (ymfK)	KT	Unclear
cydA	KT	Errors in library construction

cydC	KT	Errors in library construction
dicA	KT	Errors in library construction
purB	KT	Conditionally essential
racR	KT	Unclear
rpoE	KT	Errors in library construction
tadA	KT	Errors in library construction
ubiB	KT	Conditionally essential
ubiD	KT	Conditionally essential
wzyE	KT	Unclear
argU	P	RNA genes not considered in our analysis
argX	P	RNA genes not considered in our analysis
cysT	P	RNA genes not considered in our analysis
efp	P	Errors in library construction
ffs	P	RNA genes not considered in our analysis
glyT	P	RNA genes not considered in our analysis
hisR	P	RNA genes not considered in our analysis
kdsC	P	Polar insertions
leuU	P	RNA genes not considered in our analysis
leuW	P	RNA genes not considered in our analysis
leuZ	P	RNA genes not considered in our analysis
polA	P	Genes containing a transposon free region
priA	P	Errors in library construction
proM	P	RNA genes not considered in our analysis
serT	P	RNA genes not considered in our analysis
serV	P	RNA genes not considered in our analysis
thrU	P	RNA genes not considered in our analysis
trpT	P	RNA genes not considered in our analysis
alaS	PT	Errors in library construction
coaA	PT	Errors in library construction
coaE	PT	Errors in library construction
dnaG	PT	Errors in library construction
dnaT	PT	Unclear
folB	PT	Conditionally essential
glmM	PT	Errors in library construction
glyS	PT	Errors in library construction
groL	PT	Errors in library construction
hda	PT	Errors in library construction
ileS	PT	Errors in library construction
lptB (yhbG)	PT	Errors in library construction
nusB	PT	Polar insertions
parC	PT	Errors in library construction
prfB	PT	Errors in library construction
rho	PT	Errors in library construction
rpoD	PT	Errors in library construction
rsgA	PT	Errors in library construction
